# Preventative health, diversity, and inclusion: a qualitative study of client experience aboard a mobile health clinic in Boston, Massachusetts

**DOI:** 10.1186/s12939-017-0688-6

**Published:** 2017-11-03

**Authors:** Zoe Bouchelle, Yasmin Rawlins, Caterina Hill, Jennifer Bennet, Leonor Xochitl Perez, Nancy Oriol

**Affiliations:** 1 The Children’s Hospital of Philadelphia, Philadelphia, PA USA; 2 Columbia University College of Physicians and Surgeons, New York, NY USA; 3 Harvard Medical School, Boston, MA USA; 4 Southwestern College, Chula Vista, CA USA

**Keywords:** Mobile health, Mobile health clinic, Health disparities, Qualitative research, The family van

## Abstract

**Background:**

There are approximately 2000 mobile health clinics operating in the United States. While researchers have established that mobile health clinics can be cost effective and improve outcomes, there is scant research examining the healthcare experience on a mobile health clinic from patients’ perspectives.

**Methods:**

Data were gathered from interviews with 25 clients receiving care on a Boston-based mobile health clinic and analyzed using grounded theory methodology.

**Results:**

Emerging patterns in the data revealed three relational and three structural factors most significant to participants’ experience of care on The Family Van. Relational factors include providers who 1) Communicate understandably, 2) Create a culture of respect and inclusivity, and 3) Are diverse with knowledge of the community. Structural factors include 1) A focus on preventative health and managing chronic disease, 2) Expeditious, free, and multiple services, and 3) Location.

**Conclusions:**

The participant accounts in this report serve to expand on prior research exploring mobile health clinics’ role in patients’ healthcare, to more clearly define the most salient aspects of the mobile health clinic model for the patients they serve, and to give voice to patients too seldom heard in the academic literature.

**Electronic supplementary material:**

The online version of this article (10.1186/s12939-017-0688-6) contains supplementary material, which is available to authorized users.

## Background

In the United States, healthcare costs are high, outcomes are poor, and health disparities persist. In 2010, the U.S. spent a staggering $2.6 trillion on healthcare, while U.S. health indicators rank among the worst of high-income countries [1]. Further, racial, ethnic, and socioeconomic disparities in health and healthcare persist across a range of illnesses and services [2–4].

Mobile health clinics (MHCs) are one intervention that seeks to address these issues. There are an estimated 2000 MHCs currently operating in the U.S., receiving 6.5 million visitors annually living predominantly in medically underserved areas [5]. Research suggests that MHCs can yield impressive returns on investment, achieve good patient outcomes, and reach vulnerable populations. For example, in a 2009 study, The Family Van, a Boston-based MHC, estimated that $3,125,668 in annual costs were avoided by providing services on The Van rather than in the Emergency Department, and that annual potential life years saved by The Van services totaled $17,780,000 (at a value of $70,000 per life year) [6].

While researchers have established that MHCs have the potential to succeed in reaching medically vulnerable populations and can be cost effective [7–11], there is limited research examining patients’ experiences in such settings and what role these experiences play in patients’ healthcare. Particularly in a city like Boston, with nearly universal health insurance coverage and a dense healthcare infrastructure [12], it remains unclear why patients would choose to board a mobile clinic with relatively limited resources to receive healthcare. In a time in U.S. history during which the continued provision of health insurance under the Affordable Care Act remains uncertain [13], it is important to explore alternative and supplementary models of healthcare capable of reaching the nation’s most medically vulnerable populations.

Globally, there exists substantial need for novel healthcare delivery models to reach populations and communities with limited access to healthcare. Indeed, a recent report by the WHO and World Bank examining global access to essential health services — including family planning, antenatal care, skilled birth attendance, child immunization, antiretroviral therapy, tuberculosis treatment, and access to clean water and sanitation — found that at least 400 million people globally lacked access to at least one of these services in 2013 [14]. While there is sparse research detailing the presence and impact of MHCs globally, a 2016 review by Khanna and Narula suggests that MHCs are being employed internationally as well to reach vulnerable populations [15].

The participant accounts in this report serve to expand on prior research exploring MHCs’ role in patients’ healthcare, to more clearly define the most salient aspects of the MHC model for the patients they serve, and to give voice to patients too seldom heard in the academic literature.

## Methods

A qualitative design was chosen to explore participants’ experiences receiving health services. From June–July 2014, 25 clients were recruited by two researchers (ZB and YR) to participate after receiving care aboard The Family Van at each of the MHC’s five unique sites (Roxbury, Dudley Square, East Boston, Codman Square, Upham’s Corner). Convenience quota sampling ensured representation of the following baseline characteristics: male/female, white/non-white, ≥65/<65 years of age, and insured/uninsured. Clients were informed that participation was voluntary and would not affect their care aboard The Family Van, their responses were confidential, and that they could terminate the interview at any time. The Institutional Review Board (IRB) of the Harvard University Faculty of Medicine determined that the protocol met the criteria for exemption per the regulations found at 45 CFR 46.101(b)(2).

Interviews were conducted by two researchers (ZB and YR) in private rooms aboard The Family Van or in donated rooms in nearby community centers or businesses. Additional file 1 includes an Interview Guide with a list of questions that served to guide client interviews. A semi-structured interview approach was chosen to provide reliable, comparable qualitative data as interviews were conducted by two separate interviewers. Specific probes were used following open-ended questions where appropriate. Questions were further refined during the study. Only a portion of questions were asked during each interview; the interview question bank was large and clients were permitted to elaborate when they wished, decline to answer when they chose, and end the interview at any time.

Interviews were audio-recorded using a Zoom H1 Ultra-Portable Digital Audio Recorder and transcribed by two researchers (ZB and YR) without reference to names or other identifying characteristics. Recorded interviews ranged in length from 6.8 min to 50.9 min, for an average of 20.8 min per interview and a total of 8.7 recorded hours. Interview transcripts were subsequently uploaded to an encrypted server.

Data were analyzed by two researchers (ZB and YR) using a grounded theory approach facilitated by NVivo 10 software for Windows. Grounded theory involves iterative rounds of data collection and analysis resulting in the identification of themes grounded in the data. In this study, each researcher (ZB and YR) independently analyzed the interview transcripts noting recurrent categories and themes within and across transcripts. Regular discussions were held to compare and discuss evolving themes and categories. Coding differences were resolved by returning to interview transcripts to compare the coded quotation in question to existing quotations within a category or theme. This iterative process generated a confirmed coding structure. This process continued until no new themes emerged from the interviews. It was felt that saturation was achieved after 25 interviews. Using the confirmed coding structure, the interview transcripts were recoded generating a final dataset. Emergent themes, categorized into three structural and three relational factors most significant to participants’ experience, are described in this report using illustrative quotes.

## Results

Interviews were conducted with 25 clients receiving care on The Family Van from June–July 2014. Table 1 provides characteristics of the 25 participants in this study alongside the characteristics of clients seeking care on The Family Van in 2013. Of the 25 participants, the percentage interviewed at each site includes: Codman Square 24%, Dudley Square 24%, East Boston 28%, Save-A-Lot/Malcolm X Park 16%, and Upham’s Corner 8%. Of all of the visits to The Family Van in 2013, the percentage that occurred at each site includes: Codman Square 25%, Dudley Square 16%, East Boston 14%, Save-A-Lot/Malcolm X Park 11%, and Upham’s Corner 14%. Additional visits during 2013 occurred at Special Event sites and at previously visited sites not in service during the study period.

**Table 1:** Demographics of Interviewed Family Van Clients (25 Clients) and Family Van Client Population in 2013 (4319 Clients)

	Interviewed Clients	2013 Client Population
Gender		
Female	40%	52%
Male	60%	48%
Age		
Younger than 50	44%	42%
50–60 Years Old	16%	35%
Older than 65	40%	23%
Race or Ethnicity		
Asian	4%	12%
White (or Caucasian)	12%	21%
Hispanic	20%	23%
Black (including African-American, African and Caribbean)	36%	62%
Mixed	8%	2%
Other	16%	12%
Insurance Status		
No Insurance	12%	11%
Private Insurance	28%	23%
Public Insurance	52%	65%
Both Private and Public Insurance	8%	–

Comparison of gender, age bracket, race/ethnicity and insurance status between interviewed clients in calendar year 2013 and interviewed clients from June–July 2014

From 226 single-spaced pages of data from participant interviews, emerging patterns revealed three relational and three structural factors most significant to participants’ experience of care on The Family Van. Relational factors include providers who 1) Communicate understandably, 2) Create a culture of respect and inclusivity, and 3) Are diverse with knowledge of the community. Structural factors include 1) A focus on preventative health and managing chronic disease, 2) Expeditious, free, and multiple services, and 3) Location. Tables 2 and 3 provide salient quotes that demonstrate each of these factors. Each structural and relational factor is further described below using illustrative quotes.

**Table 2:** Valued relational aspects of care and exemplary quotations

Providers communicate understandably	“Yeah they explain it very well. To the point where I know exactly what they mean when they say what they say. Even though they probably are educated to the point where they could use big words, you know what I mean, they don’t. They make it plain, simple, and to the point so you can understand.”
	“The guy that did my HIV test, he explained it really in detail and even when I asked him more questions about like ‘Okay so if the test was positive, what would it look like?’ And he explained that to me, because I never knew.”
Providers create a culture of respect and inclusivity	“They don’t care what you look like, they don’t care what you dress like, they don’t care what you smell like, you’re always welcome in.”
	“I think The Family Van is just great with everybody, that’s why they have so many people in there. You could be black, white, you could be Spanish, you could be not Spanish, you could be, you know, anything. And they still have the same respect for everybody.”
Providers are diverse with knowledge of the community	“They understand the community because they are within our community...they understand our culture, they understand exactly what it is that we are going through. Sometimes when I try to say things and it doesn’t come out right, they already know what I’m trying to say.”
	“Why not be integrated into a holistic kind of situation? Not only just looking at your health, looking at your housing, looking at your employment, looking at your family situation…it’s a whole range that you can be plugged into when you come into a situation like The Van.”

Salient quotations demonstrating the three most valued relational factors of The Family Van identified by interviewed clients

**Table 3:** Valued structural aspects of care and exemplary quotations

Preventative health and chronic disease management	“To me the doctor’s office, they don’t tell you about preventative things. They tell you about treating things. And I want prevention, I don’t want to treat a problem, I want to prevent a problem from becoming more of a problem.”
	“I just come get my check here and so I can bring the result back to [my doctor] so I can say, ‘Over the few months that I haven’t seen you, here’s what’s been happening.”
Expeditious, free, and multiple services	“This is great. I walk in, I see one person, I see another person, the tests are right there, the results are right there, I didn’t have to go to the lab, I didn’t have to wait, I didn’t have to go upstairs with an elevator. So everything was just right there. Perfect.”
	“Like I said, we are in the ghetto. A lot of people around here don’t have money, a lot of people around here are struggling. So with it being free, it’s accessible for everybody who wants it.”
Location	“As far as it being right here, sitting right here across from Dudley Station, as crowded as Dudley Station is, it is very accessible. If it was somewhere else I don’t think it’d be that accessible, because this is the center of the transportation system.”
	“This is an area where a lot of people do need to get tested because as you look around, this is a harsh area. There’s a lot of drugs, a lot of prostitution, a lot of that type of stuff. And some people don’t feel comfortable going to the doctor’s or, don’t have the insurance to go to the doctors. So with this Van being there, they can go right there…”.

Salient quotations demonstrating the three most valued structural factors of The Family Van identified by interviewed clients

### Relational factors

#### Providers communicate understandably

One emergent theme in the dataset was the simple, direct communication that occurred between patients and providers. Many participants noted that providers on The Van communicated understandably, employing simple terms and pausing to define more complex medical terminology. One participant recalled:

Yeah they explain it very well. To the point where I know exactly what they mean when they say what they say. Even though they probably are educated to the point where they could use big words, you know what I mean, they don’t. They make it plain, simple, and to the point so you can understand. Instead of saying whatever they wanna say and you be like Uh, what does that mean? They don’t go that way. They just make it plain and simple which is the best way to do it anyway.

In contrast, another participant described his interactions in more traditional settings: “Some doctors, I always tell them, ‘Talk to me in layman’s terms. I know you went to medical school, but no medical jargon. Just talk to me — what is it? Am I going to die?’” One participant directly contrasted her experience on The Van to that in her primary care doctor’s office: “I think the quality was better [on The Family Van] because they explained things on The Van. And my doctor doesn’t really explain stuff to me.” Beyond communicating in understandable terms, participants valued providers’ directness in communication. As one participant remarked: “Right, they’re very honest. They’ll say, ‘I think you need to do this, I think you need to do that. *I can’t make you*, but I suggest that you do this and you do that.’”

Another valued aspect of communication aboard The Van was that providers appeared willing and available to answer questions and address concerns. For example, one participant noted:

The guy that did my HIV test, he explained it really in detail and even when I asked him more questions about like ‘Okay so if the test was positive, what would it look like?’ And he explained that to me, because I never knew. Because I always take em [sic] but I actually never knew what actually it looks like…And then the lady who did my diabetes test, she told me the numbers on the screen and showed me how to read it.

Many participants felt that providers also encouraged clients to ask questions about their care. As one participant described:

But they also let me know that I can ask questions. I mean, they be like, ‘You know you can ask us anything, any kind of questions you can ask and we’re here to help you. If we can answer them, we will. If we can’t, we can refer you to somebody that can.’ So it’s like the way they go about it makes you comfortable even if you are uncomfortable.

For some participants, the feeling of being able to freely ask questions of providers aboard The Van stood in contrast to their experiences with their doctors. As one participant recalled, “In the Van, I’m more comfortable. Even though I’ve known my doctor for 23 years, I’m shy with him.”

#### Providers create a culture of respect and inclusivity

A second emergent theme in the relationship between clients and providers was a perceived culture of respect and inclusivity. One participant remarked, “They treat me real respectable, real nice and they admire me, I admire them too so I don’t have no complaints with the people on The Van.” Another noted: “When I’m here I feel safe and I don’t think no one’s gonna bother me and no one bothers me and everyone respects me and I respect them and that’s the way it is.”

Not only did participants appreciate being treated with respect themselves, but many noted what they perceived as equitable treatment of others. One participant described what he/she believed to be the approach of Van providers to clients: “They don’t care what you look like, they don’t care what you dress like, they don’t care what you smell like, you’re always welcome in.” Another client communicated a similar perception of equitable treatment:

I think The Family Van is just great with everybody, that’s why they have so many people in there. You could be black, white, you could be Spanish, you could be not Spanish, you could be, you know, anything. And they still have the same respect for everybody.

Other participants expressed feeling that they received more than just the provision of health care aboard The Van. As one participant remarked, “This is business; at the same time, friendship.” A recurrent theme among participants was client-provider relationships imbued with a sense of family and community. As one participant described: “You feel like sometime you get used to know them and when you go there it’s like you meet your family sometimes.” Another participant described her experiences with providers similarly:*“*Jokey, very polite…You can just tell they have a good nature about them, by the way they’re speaking to you. Try to make you feel more like at home, rather than a visit.*”* Another echoed: “The staff is very friendly, fun, they make you laugh, and you feel like you’re going over to a family member’s house.”

When participants were asked to describe their experience of care in traditional healthcare settings, responses were mixed. While many participants described positive relationships with and expressed gratitude for their traditional healthcare providers, others described a noticeable difference between their relationships aboard The Van and those in more traditional healthcare settings. One participant stated simply: “It’s more personal [at The Van] than it is at the clinic.” Another described his/her prior visits to traditional healthcare settings, “In the past, some of the doctors were kind of cold…I think that’s the problem. When you see a doctor, and don’t have a doctor-patient relationship.”

#### Providers are diverse with knowledge of the community

A third highly valued aspect of client-provider relationships aboard The Van that emerged in the dataset were the cultural diversity and intimate knowledge of the community among Van staff. Participants highlighted the importance of The Van’s services being delivered by a staff reflecting, in many ways, the communities that they serve. In some instances, participants felt this facilitated a deeper understanding of the community that improved client-provider communication. For example, one participant commented:

They understand the community because they are within our community...they understand our culture, they understand exactly what it is that we are going through. Sometimes when I try to say things and it doesn’t come out right, they already know what I’m trying to say.

The diversity of the staff was felt to not only improve communication, but create a sense of safety and comfort as well. As one participant explained:

I feel like y’all have different diversity. So you have white people, you have black people, you have somebody that’s Spanish. So if you don’t feel comfortable because of a race issue, there’s somebody of every different race on the Van, which is good.

Extending beyond the *context* of client-provider interactions, staff diversity was perceived to influence the *content* of client-provider interactions as well. Participants felt that Van staff succeeded not just in delivering education and information but in delivering highly relevant and accessible information for the clients they serve. As one participant noted: “The quality of service y’all offer and everything is good, but the information that they provide on this Van is the number one service here on this Van.” Another participant elaborated: “You have a lot of information that you can go to to try to help you out with health insurance or social services…[The Van] has more knowledge about where to get the information.”

For many participants, the relevant information available to them through The Van extended beyond medical screenings to topics like insurance coverage and social services access. One participant explained: “…You have a lot of information that you can go to to try to help you out with health insurance (where you can apply) or social services. So [The Van has] more knowledge where to get the information.” Another participant noted feeling that The Van was not only a source of information about health and social services but approached her health and well-being in an integrated, client-centered way:

Why not be integrated into a holistic kind of situation? Not only just looking at your health, looking at your housing, looking at your employment, looking at your family situation…it’s a whole range that you can be plugged into when you come into a situation like The Van.

### Structural factors

#### Focus on preventative health and chronic disease management

The Van’s perceived focus on preventative health and managing chronic disease was an oft-repeated theme in participant interviews. Participants described The Van’s commitment to preventative health as ranging from the provision of basic healthcare screenings to what was perceived as an altogether different mindset to health and well-being from more traditional healthcare providers. One participant described frustration in seeking care at his doctor’s office, feeling that healthcare was perceived merely as treatment once a disease was diagnosed:

To me the doctor’s office, they don’t tell you about preventative things. They tell you about treating things. And I want prevention, I don’t want to treat a problem, I want to prevent a problem from becoming more of a problem. Cause my high blood pressure, my doctor wanted to put me on pills and I refused. They’re not trying to prevent it, they’re trying to treat it. To me that’s the backwards way.

Beyond preventative screenings, participants described a culture of health promotion aboard The Van, ranging from goal-setting to tracking lifestyle interventions. As one participant noted: “The Van helped me set the right benchmarks to make sure I’m active, I’m physical. What I need to do right, what I’m doing correctly, what I’m not doing correctly.”

Among those participants who had been previously diagnosed with a chronic disease, many valued The Van’s perceived commitment to prevent progression of their disease. While participants acknowledged that The Van did not have the resources to provide prescriptions or medically treat their disease, they viewed the frequency with which they were able to visit The Van as a key component in managing their disease that they were often unable to access in more traditional healthcare settings. As one participant described:

That’s another reason why I come on the Van a lot, because I have to wait three or four months before I see my primary care doctor. So within that period of time I wanna make sure I’m still doing alright along the way before I do see him.

In addition to remaining apprised of their own health status with frequent screenings and check-ins at The Van, many participants noted that as result of their relationship with The Van, they were able to provide more information to their doctors during the visits that they did have. Indeed, some participants used check-ins at The Van as a way to generate additional data points that could then be communicated to their doctors with the goal of improving their care. As one participant noted: “I just come get my check here and so I can bring the result back to [my doctor] so I can say, ‘Over the few months that I haven’t seen you, here’s what’s been happening.”

#### Expeditious, free, and multiple services

A second highly valued aspect of The Van’s healthcare structure that emerged during the interviews were expeditious, free, and multiple services. Despite the majority of clients receiving care on The Family Van being insured, cost still emerged as a highly valued aspect of The Van’s service. One participant described the economic challenges in his/her community and the value of a service like The Van: “Like I said, we are in the ghetto. A lot of people around here don’t have money, a lot of people around here are struggling. So with it being free, it’s accessible for everybody who wants it.”

In addition to low-cost service, the relatively expedient delivery of The Van’s services emerged as a valued aspect of care. One participant described his experience of entering The Van, getting tests, and being informed of his results:

This is great. I walk in, I see one person, I see another person, the tests are right there, the results are right there, I didn’t have to go to the lab, I didn’t have to wait, I didn’t have to go upstairs with an elevator. So everything was just right there. Perfect.

Another participant expressed a similar experience: “They were able to take care of my needs very promptly…and help me understand that I did not have HIV. If it wasn’t for the bus, I don’t think I would have known that today.”

Many participants contrasted their experiences accessing timely services on The Van with their experiences in more traditional healthcare settings. As one client described: “At the clinic, I mean, I go in, and I gotta wait, and fill out forms and stuff just for a simple thing like a blood pressure.” For many participants, this expediency in service extended beyond the relative speed of receiving a screening and its result to the ability to access care without a prior appointment. One client contrasted his experience attempting to receive care at a hospital to his experience aboard The Van:

You want to go to a hospital but you have to make an appointment. This right here you don’t have to wait six months for your appointment, you can just go right in there and really get a head start and know what’s going on…

For many participants, the ease in accessing care on The Van was bolstered by the ability to receive multiple screenings from a single access point. As one client described: “It’s fast, easy, and you’re getting the basic services done right then and there...the flexibility of just opening the door and getting four to five different check-ups done, is very unique.”

#### Location

A third highly valued aspect of The Van’s healthcare structure that emerged during the interviews was the location. Many participants valued the fact that The Van was often parked in close proximity to their residence, to hubs of public transportation, and en route to their day-to-day activities.

As one participant explained succinctly when asked why he/she came to The Van for care: “Convenience. It was right down the street from me.” Another participant noted its location on his/her daily route:

Let’s say, ‘Hey I have to go to work or I have to go teach or I have to go to the grocery store but I wanna go to the hospital’...The convenience is like ‘Hey I’m going to the grocery store right here, Brothers [Supermarket], and The Van’s right here.’

Commenting on The Family Van’s accessibility via public transport, one participant explained:

As far as it being right here, sitting right here across from Dudley Station, as crowded as Dudley Station is, it is very accessible. If it was somewhere else I don’t think it’d be that accessible, because this is the center of the transportation system.

The Family Van’s ability to frequent multiple sites was valued by many participants. As one stated: “I think it’s great that you are all over the city, that you drive to the different sites. Because in the inner city, not everybody has a car.” Participants noted the many barriers to seeking healthcare, ranging from cost to convenience to transportation. The Van was able to address and overcome many of these barriers, as one participant described:

This is an area where a lot of people do need to get tested because as you look around, this is a harsh area. There’s a lot of drugs, a lot of prostitution, a lot of that type of stuff. And some people don’t feel comfortable going to the doctor’s or, don’t have the insurance to go to the doctors. So with this Van being there, they can go right there.

## Discussion

This report describes client experiences from a set of 25 participants seeking health services aboard The Family Van. While prior studies have focused on MHCs that provide tailored care for specific populations — including the elderly, asthmatic children, veterans, and homeless individuals [16–19] — this study examines the valued aspects of an MHC providing a broad range of preventative services to an urban U.S. population. From 226 single-spaced pages of data from participant interviews, three relational and three structural factors emerged as most significant to participants’ experience of care on The Family Van. Relational factors include providers who 1) Communicate understandably, 2) Create a culture of respect and inclusivity, and 3) Are diverse with knowledge of the community. Structural factors include 1) A focus on preventative health and managing chronic disease, 2) Expeditious, free, and multiple services, and 3) Location. The findings in this report expand on prior research exploring MHCs’ role in patients’ healthcare, more clearly define the most salient aspects of the MHC model for the patients they serve, and give voice to patients too seldom heard in the academic literature.

Prior research on barriers to healthcare access suggests that patients living in medically underserved communities face structural and logistical barriers to care including inadequate health insurance and inaccessible transportation. Poor access to preventative healthcare and a primary care provider have been associated with worse health outcomes, as well as racial, ethnic, and socioeconomic health and healthcare disparities across a range of illnesses and services [3, 20, 21]. This lack of access to basic health services extends internationally. A 2015 report by the WHO and World Bank found that at least 400 million people globally lacked access to at least one of seven basic health services in 2013 [14].

By nature of their model of care, MHCs have the capacity to address many of these barriers and prior research on MHCs support their ability to do so [22, 23]. For example, Alexy and Elnitsky [16] describe a rural MHC serving elderly patients in the U.S. with difficulty obtaining care due to illness, problems with transportation, and financial factors; this MHC parked at three senior centers and four well-utilized community sites, increasing breast and cervical cancer screenings, immunization rates, and participant knowledge of available primary care while decreasing emergency room utilization. Bollinger, Morphew, and Mullins [19] and Jones et al. [24] describe The Breathmobile program, a mobile asthma clinic in the U.S. that provides free care to underserved children; this MHC parked at neighborhood schools, Head Start sites, and community centers, decreasing emergency room visits and increasing symptom free days for participants at low-cost. Khanna and Narula highlight a number of MHCs working internationally in their 2016 review published in the International Journal of Healthcare Management [15]. The qualitative findings in this report — that participants experience fewer cost and transportation barriers when seeking care aboard The Family Van — complement this prior quantitative research.

Beyond its low cost and geographic convenience, participants described The Van as playing a complementary role to their traditional healthcare provider, both for acute and chronic care. While some participants reported using The Family Van as an alternative to the emergency department or as a bridge to other forms of healthcare, the majority reported using the MHC as a complement; it served as a more frequent and accessible link to the healthcare system. For participants facing a chronic disease, many used The Family Van as a way to track their progress in order to provide their primary physician with more information on their blood pressure or diabetes control. As one participant noted:

That’s another reason why I come on the Van a lot, because I have to wait three or four months before I see my primary care doctor. So within that period of time I wanna make sure I’m still doing alright along the way before I do see him.

While many participants had access to glucometers and sphygmomanometers at home, they preferred visiting The Family Van because they trusted staff to take accurate readings and provide guidance and support. Armed with more “data points” regarding their health, participants were able to provide their primary doctors with a week-to-week report of their health — rather than merely the results of an annual visit — to guide their recommendations and management decisions. This finding is particularly salient given that many disparities related to chronic disease grew larger in 2014 [3] and there is increasing evidence for the value of preventative health services in decreasing healthcare costs and improving health outcomes [25].

Research has also shown that patients living in underserved communities have greater difficulty accessing care due to a number of relational barriers including cultural differences, poor communication, a perceived absence of patient-centered care, and a lack of diversity among medical professionals [26–29]. Poor communication between patients and providers has been known to lead to worse outcomes, while clear communication and strong relationships can lead to better treatment adherence and health status [30, 31]. Lack of cultural competence is increasingly being implicated in health and healthcare disparities, and a lack of diversity among medical professionals is a persistent issue [32, 33].

The Family Van, and similarly designed MHCs, have the capacity to address many of these relational and logistical barriers, and prior research on MHCs support their ability to do so. For example, Carmack [34] describes how a rural mobile clinic in Appalachia delivers medical services in a safe, non-hierarchical space, thereby creating a comfortable, trusting environment for patients. Guruge, Hunter, Barker, McNally, and Magalhaes [35] describe how an MHC in Toronto, Canada delivers culturally competent, community-based care for immigrant women. Similarly, participants in this report emphasized clear communication and transparency in the clinical encounter. Participants noted that although Van providers had the education to describe disease and treatment in complex terms, they used terms that participants could understand. As one participant recalled:

Yeah they explain it very well. To the point where I know exactly what they mean when they say what they say. Even though they probably are educated to the point where they could use big words, you know what I mean, they don’t. They make it plain, simple, and to the point so you can understand. Instead of saying whatever they wanna say and you be like Uh, what does that mean? They don’t go that way. They just make it plain and simple which is the best way to do it anyway.

Providers were also noted to engage patients in the process and encourage questions creating a bidirectional dialogue. Further, the close relationships that participants reported between themselves and providers, due in part to providers’ knowledge of the community, improved their healthcare experience and contributed to their reasons for continuing to visit. As one participant described the staff, “They don’t care what you look like, they don’t care what you dress like, they don’t care what you smell like, you’re always welcome in.” Another client echoed:

I think The Family Van is just great with everybody, that’s why they have so many people in there. You could be black, white, you could be Spanish, you could be not Spanish, you could be, you know, anything. And they still have the same respect for everybody.

In short, participants describe a welcoming, patient-centered approach to care — an approach to healthcare which has been linked to better treatment adherence and increased health literacy [31, 36].

While the strengths of this study are its detailed descriptions of the experience of clients aboard an MHC, it has limitations. The study participants were a self-selected sample, which may skew the data toward more positive or negative accounts of MHC experiences. Additionally, the researchers’ presence during data gathering, which is often unavoidable in qualitative research, can affect participants’ responses. While every effort was made to ensure privacy, participants were often interviewed in private rooms aboard The Van and in nearby community sites, which may have influenced their responses. Though open-ended questions were used in the interview process, our semi-structured approach introduced several themes to our interviews which were likely over-represented compared to participant-generated themes.

## Conclusions

This report examines the most valued aspects of an MHC from the perspective of the clients it serves. As might be expected, participants highlighted cost and location as valued aspects of the MHC. Less predictably, however, participants emphasized the importance of a diverse set of Van providers who played a complementary role to their primary care provider. This suggests the value of the MHC model lies not only in its cost and convenience but in its ability to meet patients where they are, in their communities, manned by professionals from within the community. It also suggests that MHCs may have the capacity to play a more significant role in providing frequent healthcare access to medically underserved populations and serve a complementary role to primary care providers practicing in brick and mortar, more resourced settings. This has implications not only in the U.S., but for every country striving to provide basic health services to its citizens. While the limitations of MHCs are many, this report joins a small but growing body of literature suggesting that they have the potential to provide low-cost, high-value, preventative health and education to a larger population in the U.S. and around the world.

## Additional file

Additional file 1: Interview Guide. (DOCX 15 kb)

## References

[1] Health Care Costs: A Primer. https://kaiserfamilyfoundation.files.wordpress.com/2013/01/7670-03.pdf. Accessed 10 April 2017.

[2] Nelson A (2002). Unequal treatment: confronting racial and ethnic disparities in health care. J Natl Med Assoc.

[3] National Healthcare Quality & Disparities Report.2014 https://www.ahrq.gov/research/findings/nhqrdr/nhqdr14/index.html. Accessed 10 April 2017.

[4] Health disparities and inequalities report. Morbidity and Mortality Weekly Report 2013; 62 Suppl 3:1–187.

[5] Hill C, Powers B, Jain S, Bennet J, Vavasis A, Oriol N (2014). Mobile health clinics in the era of reform. Am J Manag Care.

[6] Oriol NE, Cote PJ, Vavasis AP, Bennet J, Delorenzo D, Blanc P, Kohane I (2009). Calculating the return on investment of mobile healthcare. BMC Med.

[7] Song Z, Hill C, Bennet J, Vavasis A, Oriol NE (2013). Mobile clinic in Massachusetts associated with cost savings from lowering blood pressure and emergency department use. Health Aff.

[8] Nuttbrock L, Rosenblum A, Magura S, Mcquistion H (2003). Broadening perspectives on mobile medical outreach to homeless people. J Health Care Poor Underserved.

[9] Diaz-Perez MJ, Farley T, Cabanis CM (2004). A program to improve access to health care among Mexican immigrants in rural Colorado. J Rural Health.

[10] Hill C, Zurakowski D, Bennet J, Walker-White R, Osman JL, Quarles A, Oriol N (2012). Knowledgeable neighbors:a mobile clinic model for disease prevention and screening in underserved communities. Am J Public Health.

[11] Edgerley LP, El-Sayed YY, Druzin ML, Kiernan M, Daniels KI (2007). Use of a community mobile health van to increase early access to prenatal care. Matern Child Health J.

[12] Almost Universal Coverage in Massachusetts: Leading the Nation in Health Insurance – MassBudget. http://www.massbudget.org/report_window.php?loc=ACS2014.html. Accessed 10 April 2017.

[13] Harvey H, Banthin J, Chirico C. Congressional Budget Office Cost Estimate: American Health Care Act https://www.cbo.gov/system/files/115th-congress-2017-2018/costestimate/americanhealthcareact.pdf. Accessed 10 April 2017.

[14] Tracking universal health coverage (2015). First global monitoring report.

[15] Khanna A, Narula S. Mobile health units: mobilizing healthcare to reach unreachable. International journal of healthcare management [serial online] April 2016;9(1):58-66. Available from: business source complete, Ipswich, MA.

[16] Alexy BB, Elnitsky C (1998). Rural mobile health unit: outcomes. Public Health Nurs.

[17] Oboler SKA (1983). Mobile internal medicine clinic. Arch Intern Med.

[18] Liebman J, Lamberti MP, Altice F (2002). Effectiveness of a mobile medical van in providing screening services for STDs and HIV. Public Health Nurs.

[19] Bollinger ME, Morphew T, Mullins CD (2010). The Breathmobile program: a good investment for underserved children with asthma. Ann Allergy Asthma Immunol.

[20] Fiscella K, Franks P, Doescher MP, Saver BG (2002). Disparities in health care by race, ethnicity, and language among the insured. Med Care.

[21] Racial and Ethnic Disparities in Access to and Quality of Health Care. http://www.rwjf.org/en/library/research/2007/09/racial-and-ethnic-disparities-in-access-to-and-quality-of-health.html. Accessed 10 April 2017.

[22] Kahn RH, Moseley KE, Thilges JN, Johnson G, Farley TA (2003). Community-based screening and treatment for STDs: results from a mobile clinic initiative. Sex Transm Dis.

[23] Reguero W, Crane M (1994). Project MotherCare: one hospital's response to the high perinatal death rate in new haven. CT Public Health Report.

[24] Jones C, Clement L, Morphew T, Hanley-Lopez J, Kwong K, Lifson F, Opas L, Guterman J (2007). Achieving and maintaining control of asthma with regular participation in an urban pediatric disease management program: the Breathmobile® program. J Allergy Clin Immunol.

[25] Maciosek MV, Coffield AB, Flottemesch TJ, Edwards NM, Solberg LI (2010). Greater use of preventive services in U.S. health care could save lives at little or no cost. Health Aff.

[26] Jupka KA, Weaver NL, Sanders-Thompson VL, Caito NM, Kreuter MW (2008). African American adults’ experiences with the health care system: in their own words. Journal of health disparities research. Practice.

[27] LaVeist TA, Nickerson KJ, Bowie JV (2000). Attitudes about racism, medical mistrust, and satisfaction with care among African American and white cardiac patients. Medical Care Research Review.

[28] Ngo-Metzger Q, Massagli MP, Clarridge BR, Manocchia M, Davis RB, Lezzoni LI, Phillips RS (2003). Linguistic and cultural barriers to care. J Gen Intern Med.

[29] O’Malley AS, Forrest CB (2003). Beyond the examination room: primary care performance and the patient-physician relationship for low-income women. J Gen Intern Med.

[30] Saha S, Arbelaez JJ, Cooper LA (2003). Patient-physician relationships and racial disparities in the quality of health care. Am J Public Health.

[31] Schneider J, Kaplan SH, Greenfield S, Li W, Wilson IB. Better physician-patient relationships are associated with higher reported adherence to antiretroviral therapy in patients with HIV infection. J Gen Intern Med. 2004;19:1096–103. 10.1111/j.1525-1497.2004.30418.x PMC1494791 15566438

[32] Betancourt JR, Green AR, Carrillo JE, Park ER. Cultural Competence And Health Care Disparities: Key Perspectives And Trends. Health Aff. 2005;24(2):499–505. doi:10.1377/hlthaff.24.2.499. 10.1377/hlthaff.24.2.499 15757936

[33] Cohen JJ, Gabriel BA, Terrell C. The Case For Diversity In The Health Care Workforce. Health Aff. 2002;21(5):90-102. doi:10.1377/hlthaff.21.5.90. 10.1377/hlthaff.21.5.90 12224912

[34] Carmack HJ. “What happens on the van, stays on the van”: the (re)structuring of privacy and disclosure scripts on an Appalachian mobile health clinic. Qual Health Res. 2010;20:1393–405. 10.1177/1049732310372618 20530401

[35] Guruge S, Hunter J, Barker K, Mcnally MJ, Magalhães L. Immigrant women’s experiences of receiving care in a mobile health clinic. J Adv Nurs. 2010;66:350–9. 10.1111/j.1365-2648.2009.05182.x 20423418

[36] Levinson W, Lesser CS, Epstein RM. Developing physician communication skills for patient-centered care. Health Aff. 2010;29:1310–8. 10.1377/hlthaff.2009.0450 20606179

