# Supplementary material for: Preventative health, diversity, and inclusion: a qualitative study of client experience aboard a mobile health clinic in Boston, Massachusetts
Source: Int J Equity Health. 2017 Nov 3;16:191. doi: 10.1186/s12939-017-0688-6 (PMC5670702; doi:10.1186/s12939-017-0688-6)
Supplement: Additional file 1: — Interview Guide. (DOCX 15 kb) [file 12939_2017_688_MOESM1_ESM.docx]

| **Additional file 1.  Interview Guide** |
| --- |
| In what year were you born? |
| What gender do you identify as? |
| What race or ethnicity do you identify with? |
| What languages do you speak? |
| What language do you speak at home? |
| Do you have health insurance? What kind? |
| What medical services do you receive on The Van? |
| What non-medical services do you receive on The Van? |
| Is this your first time on The Van? If yes, what were you thinking when you first saw The Van? What were you thinking when you decided to walk on? If no, what were you thinking when you first saw The Van? What were you thinking when you decided to walk on? Why did you choose to come back? |
| How long have you been coming to The Van? How frequently or how many times have you been back? |
| How would you describe your experiences on The Van? |
| What do you think about the accessibility of The Van? |
| What do you think about the location of The Van? What do you think the weekly schedule? What do you think about the waiting time? What did you think about there being no cost? What do you think about the length of your visit? |
| When you’re on The Van, do you feel like you have enough time to talk about your concerns? |
| How would you describe your relationship with the staff on The Van? |
| Do you feel comfortable on The Van? Why or why not? |
| Do you feel that you are treated with respect on The Van? Can you tell me more about that? |
| Do you feel the staff on The Van explains things to you in terms you understand? Can you tell me more about that? |
| Do you feel the staff on The Van encourage you to ask questions? Can you tell me more about that? |
| Do you feel that you receive enough information about your concerns while on The Van? Can you tell me more about that? |
| When you’re on The Van, who do you feel makes the final decisions on your healthcare? |
| How well do you think The Van understands you? How well do you think The Van understands your community? |
| I’d like to understand what you’re thinking and how you feel when you’re on The Van. So just say the first word or phrase that comes to mind to complete this sentence. When I’m sitting on the Van, I feel  ______. |

| **Additional file 1.  Interview Guide Continued** |
| --- |
| What do you think about the level of privacy on The Van? |
| What is your favorite aspect of The Van? |
| Is there anything that your doctor does that you would like to see The Van do? |
| Are there any suggestions you would make to change The Van? |
| Have you recommended The Van to other people? Why or why not? |
| Has coming to The Van: Changed the way you think about your health? Changed your knowledge about your health? Changed your health? Changed the way you interact with your healthcare provider? |
| Where did you or do you go to receive healthcare services besides The Van? If you do go somewhere else, why do you go both places? |
| How would you describe your experiences with your other healthcare provider? |
| What do you think about the accessibility of your other healthcare provider? What do you think about the location?  What do you think the weekly schedule? What do you think about the waiting time? What did you think about the cost? What do you think about the length of your visit? Do you feel like you have enough time to talk about your concerns? |
| How would you describe your relationship with your other healthcare provider? |
| Do you feel comfortable with your other healthcare provider? Why or why not? |
| Do you feel that you are treated with respect? Can you tell me more about that? |
| Do you feel your other healthcare provider explains things to you in terms you understand? Can you tell me more about that? |
| Do you feel your other healthcare provider encourages you to ask questions? Can you tell me more about that? |
| Do you feel that you receive enough information about your concerns while with your other healthcare provider? Can you tell me more about that? |
| Do you feel like all your needs are served when you’re with your other healthcare provider? How so or why not? |
| When you’re with your other healthcare provider, who do you feel makes the final decisions on your healthcare? |
| I’d like to understand what you’re thinking and how you feel when you’re at your other healthcare provider. So just say the first word or phrase that comes to mind to complete this sentence. When I’m sitting [at my other healthcare provider’s office], I feel ______. |
| How well do you think your other healthcare provider understands you? How well do you think your other healthcare provider understands your community? |
| Do you recommend your other healthcare provider to other people? Why or why not? |
| What is your favorite aspect of your other healthcare provider? |
| Is there anything that The Van does that you would like to see your other healthcare provider do? |
| How does your experience on The Van compare to your experience in other healthcare settings? |
